# Giant Uterine Tumor Compatible With Sarcoma

**DOI:** 10.7759/cureus.80101

**Published:** 2025-03-05

**Authors:** Elias Gallardo-Navarro, Alfredo Saad Ganem, Adriana Margarita Acosta Blanco, Lucia Muradas Gil, Francisco M Garcia Rodriguez

**Affiliations:** 1 General Surgery, Hospital Español, Mexico City, MEX; 2 Gynecology and Obstetrics Service, Hospital Español, Mexico City, MEX; 3 Oncologic Surgery, Hospital Español, Mexico City, MEX

**Keywords:** abdominal mass, abdominal radical hysterectomy, metastasis, sarcoma, uterine tumor

## Abstract

Uterine sarcomas are a heterogeneous group of very rare tumors of mesenchymal origin, accounting for approximately 4% of malignant tumors of the uterus. These represent a heterogeneous group of neoplasms that are classified by histology in order of frequency as leiomyosarcoma, endometrial stromal sarcoma, and carcinosarcoma or mixed mullerian tumor. Even in its early stages, uterine sarcoma has a bad prognosis and is a very rare disease that is difficult to identify and typically detected by anatomopathological studies. They are a type of cancer derived from the mesenchyme or connective tissue of the uterus that have a wide variety of histological aspects and biological activity, which makes their classification difficult. A 78-year-old woman reported experiencing abdominal pain, adynamia, and asthenia along with an increase in abdominal volume about six months ago. A physical examination revealed this increase, and a surgical procedure involving radical hysterectomy with peritoneal implants and pelvic lymph node dissection was chosen. The patient was transferred to the intensive care unit due to hypovolemic shock secondary to abdominal decompression and intraoperative hemorrhage, with a surgical product of 6.115 kilos. The patient had an adequate clinical evolution and was discharged one week later from the hospital for adjuvant treatment with chemotherapy and radiotherapy. However, after three weeks of follow-up, she died due to disease progression. Treatment recommendations for patients with uterine sarcoma are based on the results of retrospective studies, which makes clinical management difficult.

## Introduction

Uterine sarcomas are a heterogeneous group of very rare tumors of mesenchymal origin, accounting for approximately 4% of malignant tumors of the uterus [1]. These represent a heterogeneous group of neoplasms that are classified by histology in order of frequency as leiomyosarcoma (LMS), endometrial stromal sarcoma (ESS), and carcinosarcoma or mixed mullerian tumor [2]. Sarcomas occur in older women (age at diagnosis is usually 62-67 years), the risk is twofold higher in black women compared to white women [3]. In the literature, approximately 0.3%, an occult uterine sarcoma was diagnosed, when the indication is for a suspected uterine myoma, with ranges of up to one in 256 to one in 332 patients undergoing hysterectomy or myomectomy, improving the detection of patients at risk for sarcoma before each surgery is of utmost importance to plan the surgical technique [4]. These tumors are composed of mesenchymal tissue and simple (homogeneous) and complex sarcomas have been distinguished, including homologous (derived from uterine tissue), heterologous (containing tissue not normally found in the uterus) and mixed forms (composed of a squamous or glandular epithelial component, benign or malignant, and a mesenchymal component, always malignant) [5]. Diagnosis and pathological classification of uterine sarcomas is a challenge. The most frequent subtypes are LMS followed by ESS and undifferentiated uterine sarcoma (UUS). Histological grade deeply impacts prognosis and clinical behavior. While high-grade ESSs (HG-ESSs) are generally highly aggressive, low-grade ESSs (LG-ESSs) have an indolent behavior. LG-ESSs have a five-year survival rate and a five-year disease-specific survival of 80%-100% [6]. Currently, there are no specific symptoms associated with sarcomas of the uterus, but it is suspected when there is rapid growth of the uterus in hypoestrogenic postmenopausal women, unlike carcinomas, the clinic of the disease may be with abnormal uterine bleeding, abdominal distension or compressive symptoms, sometimes they are diagnosed in patients who are treated surgically by diagnosis of uterine myomatosis, surgery (hysterectomy and adnexectomy) is essential in the treatment [7]. The histologic grade of low-grade tumors frequently has intravascular growth and infiltrating borders. However, they rarely metastasize, unlike high-grade tumors, which are infiltrating, with accentuated nuclear atypia, and in these tumors, metastases are more frequent. The International Federation of Gynecology and Obstetrics (FIGO) classifies them as stage I, when the tumor is limited to the uterus; stage II, the tumor extends to the pelvis; stage III, the tumor invades the abdominal tissues; and stage IV, tumor invades the bladder, rectum or both, or metastases to distant sites [8,9]. Appropriate classification of uterine sarcomas is essential for accurate selection of adjuvant therapy, the mainstay of treatment for LMS and ESS is surgical resection with total abdominal hysterectomy and bilateral salpingo-oophorectomy with or without lymph node sampling, the role of adjuvant therapy following surgical resection of LMS is controversial and generally reserved for advanced or recurrent disease [9,10]. We report the following case of a patient with a notorious enlargement of the abdomen with suspicion of uterine myomatosis of large elements that, when the surgery was performed, a large tumor with peritoneal implants compatible with sarcoma was observed. A review of the literature regarding the histopathological diagnosis, treatment, and prognosis of this condition is performed. This is a representative case report of an advanced type of uterine sarcoma cancer in an elderly patient, who, despite adequate surgery, the advanced stage of the disease decreased survival.

## Case presentation

**Figure 1:**
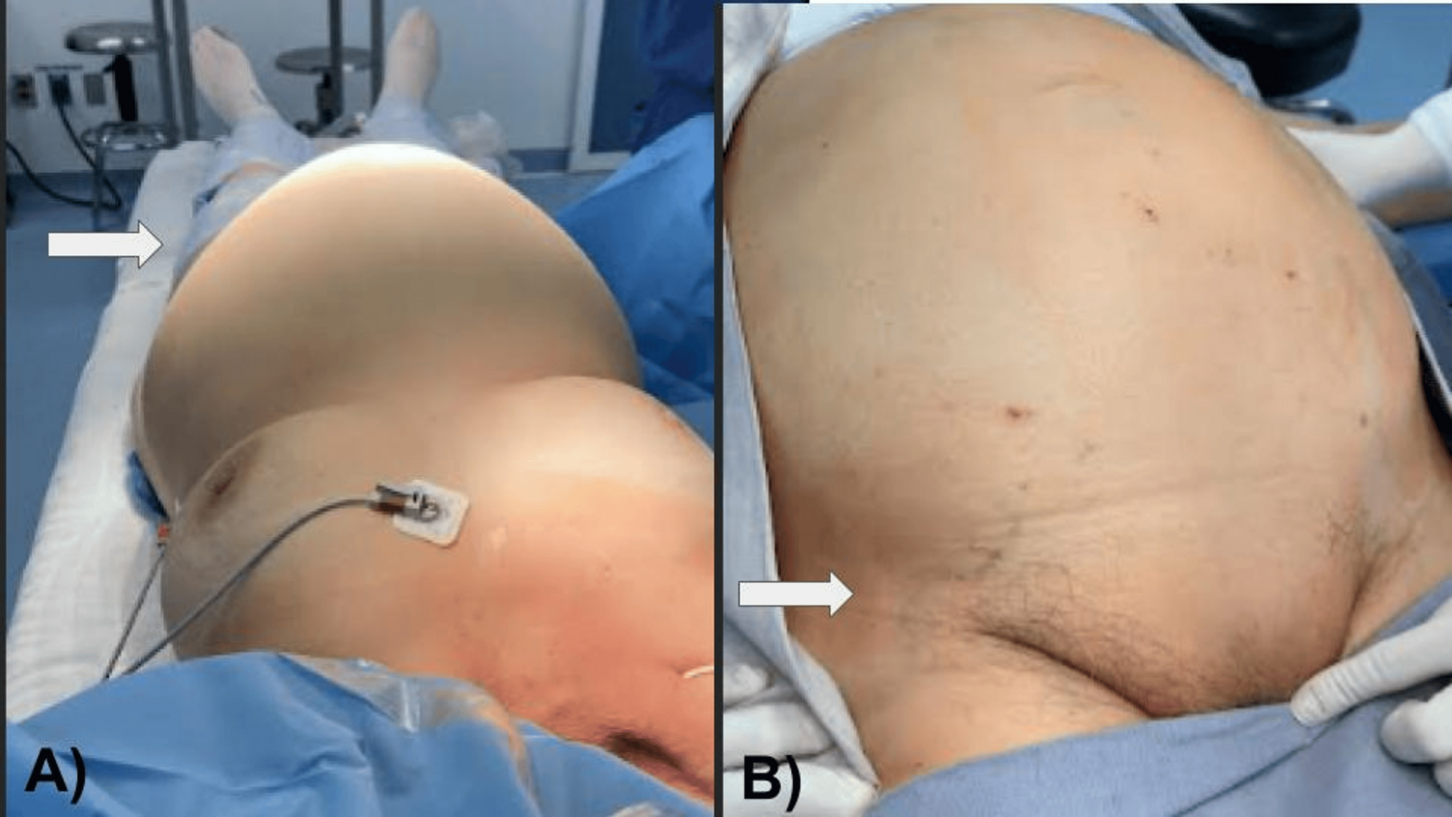
(A) Volume increase of the abdominal region is observed (arrow). (B) Collateral venous network secondary to intra-abdominal tumor compression is observed (arrow).

**Figure 2:**
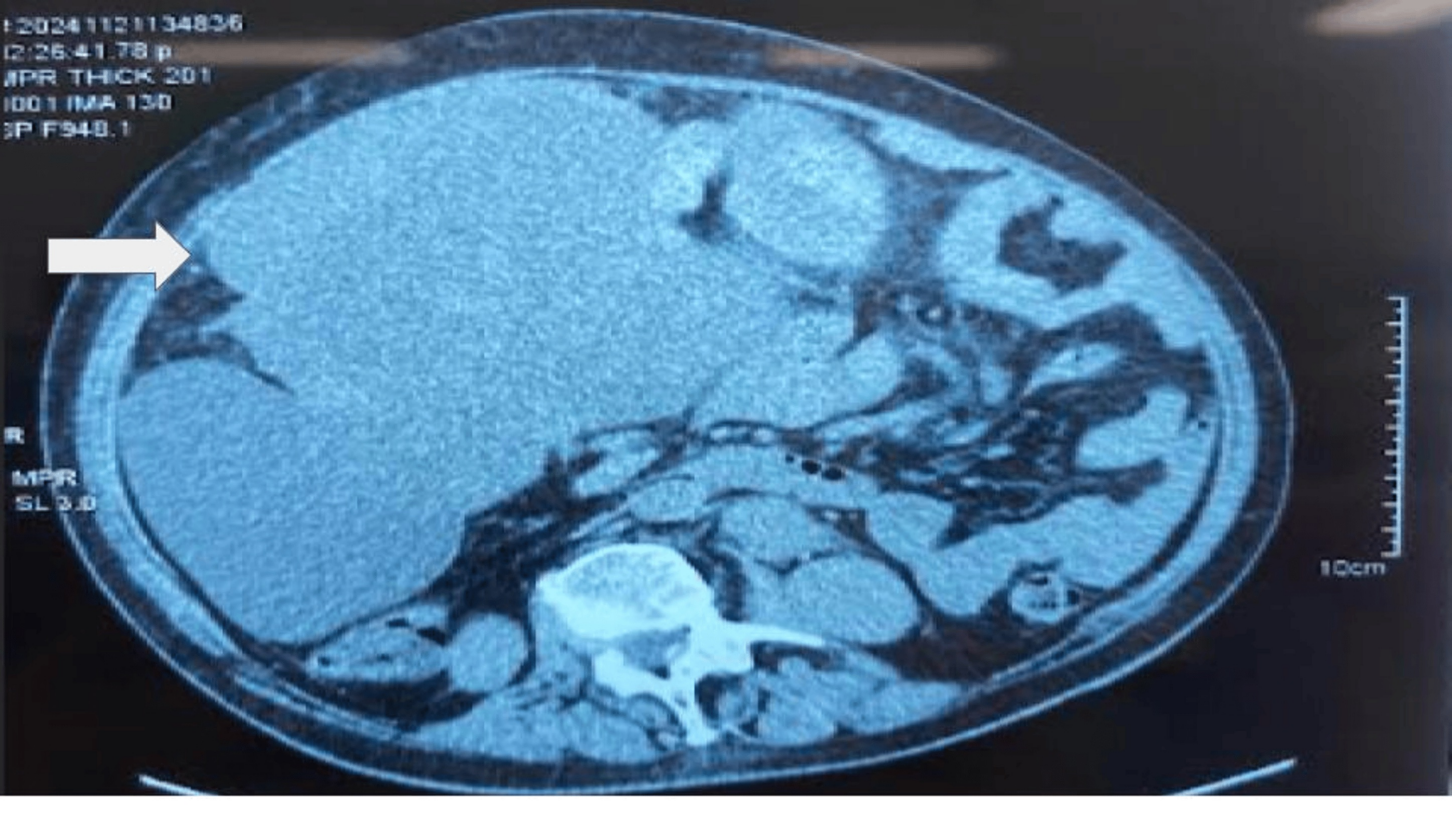
Axial MRI scan showing a mass occupying the entire space of the abdominal cavity in function of the uterine body and adnexa that displaces the viscera toward the right parietocolic slide (indicated by the arrow) and the presence of peritoneal implants.

**Figure 3:**
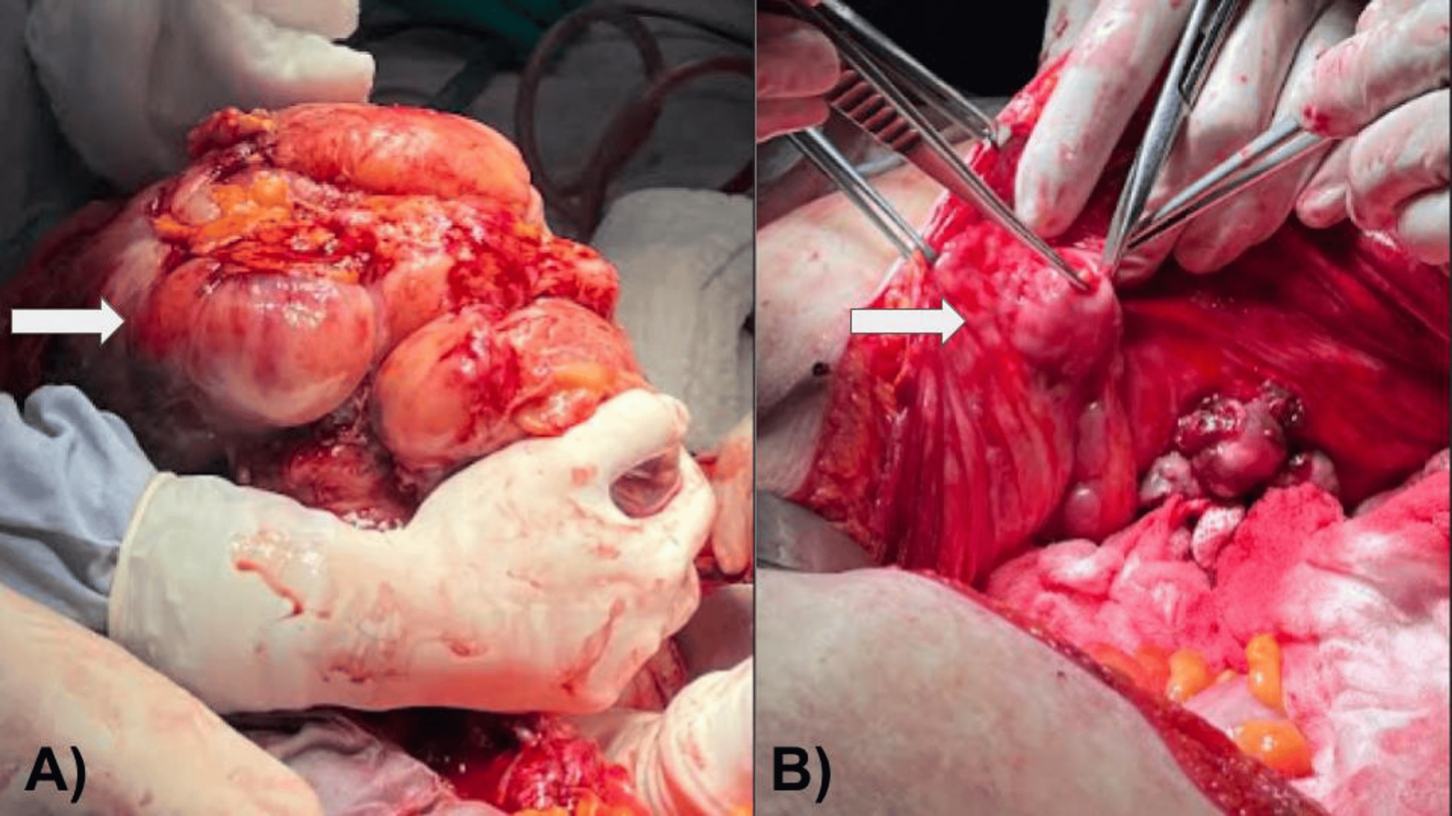
(A) En bloc resection of tumor involving uterus, ovaries, and large peritoneal implants (arrow). (B) Peritoneal implants are observed (arrow).

**Figure 4:**
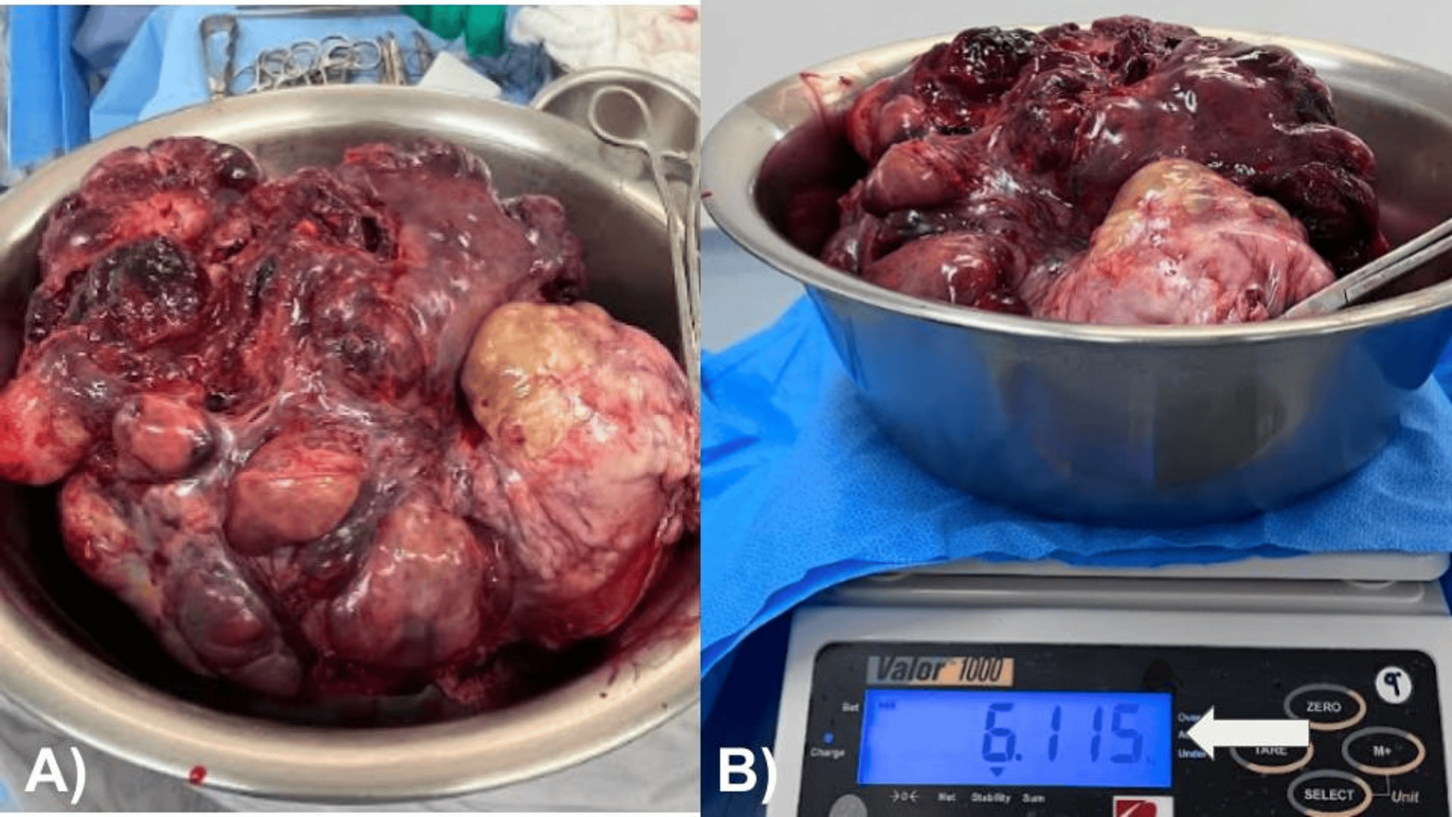
(A) Macroscopic image of the en bloc resection of the tumor. (B) Weight 6,115 kg (arrow).

A 78-year-old female with no history of significance for the current disease relates to an increase in volume in the abdominal region since approximately six months ago. A few days prior to the medical consultation, she refers to adynamia, asthenia, and abdominal pain, constipation of three days of evolution, reason for consultation. During the physical examination, it is evident the increase in volume at the expense of adipose panniculus in the abdomen (Figures 1A, 1B). In addition to presenting bilateral collateral venous network in the inguinal region secondary to increased intra-abdominal volume, the abdomen feels hard to the touch, there is hypoperistalsis and reduced mobility, there are no signs of ascites or peritoneal irritation, there is no bleeding during the gynecological examination, the parametrium is occupied by mass, and the cervix is hard and stony. General laboratories were requested, with relevant results of anemia with hemoglobin 9 g/dL, negative tumor markers, magnetic resonance imaging was requested, thinking in uterine myomatosis of large elements (Figure 2) and a mass was observed that occupies the entire space of the abdominal cavity depending on the uterine body and annexes that displaces viscera to the right parietocolic slide (indicated by the arrow) and the presence of peritoneal implants. For all these reasons, a surgical procedure was decided which consisted of radical hysterectomy with peritoneal implants, pelvic lymph node dissection and adhesiolysis of intestinal loops (Figures 3A, 3B). R2 cytoreduction was performed, with residual macroscopic disease due to the presence of abundant peritoneal implants and bowel loops involvement. The patient was transferred to the intensive care unit due to hypovolemic shock secondary to abdominal decompression and intraoperative bleeding, with surgical product weighing 6.115 kilos (Figures 4A, 4B), compatible with uterine sarcoma, stage IVA. During the postoperative period, the patient had an adequate clinical evolution and was discharged from the hospital one week later to start chemotherapy and adjuvant radiotherapy by the medical oncology service. However, due to disease progression, she died three weeks after her discharge.

## Discussion

Uterine sarcomas are a rare, heterogeneous and aggressive group of neoplasms, frequently leading to dissemination and early death [10,11]. There are few epidemiological studies to identify risk factors. On the other hand, the only etiological factor documented in 10% to 25% of these malignant neoplasms is the history of pelvic radiotherapy. It has also been related to the use of tamoxifen for the treatment of breast cancer. Subsequently, increases were also observed with the administration of tamoxifen to prevent breast cancer in women with an increased risk of breast cancer, possibly a consequence of the estrogenic effects of tamoxifen on the uterus [10]. Imaging studies to diagnose uterine sarcomas include evaluation of the appearance of the uterine tumor mass to differentiate it from other pelvic tumors. They are used to evaluate the depth of invasion, degree of infiltration to adjacent organs, lymph node involvement, and the presence of other pelvic metastases. Ultrasound is the diagnostic procedure of choice to assess the uterus. MRI offers high diagnostic sensitivity and specificity, computed tomography, to confine abdominal masses, assess nodal invasion and organ infiltration, and positron emission computed tomography (PET-CT) provides detailed data on primary staging with limited sensitivity for nodal involvement [12-14]. The determination of carbohydrate antigen 125 (Ca125) can be elevated in cases of disseminated disease, but in early stages its usefulness is controversial. Also, the levels of lactate dehydrogenase isoenzyme (LDH-3) and the lymphocyte/neutrophil ratio may be elevated in patients with uterine sarcomas, so their determination could be helpful in diagnosis and although more studies are needed, the results are promising, liquid biopsy with biomarker determination (RNA, DNA) is the future of uterine sarcoma diagnosis, but its performance should be reserved, at present, in the context of clinical trials [15-17]. Treatment is based on staging, abdominal hysterectomy with bilateral salpingo-oophorectomy, selective pelvic and para-aortic lymphadenectomy and evaluation of adjuvant chemotherapy and radiotherapy. For carcinosarcomas, the greater possibility of intra-abdominal and retroperitoneal metastasis must be considered than LMSs, because the lymph node involvement is greater in carcinosarcomas, LMSs have a low risk of occult lymph node disease from 3.5% to 11%. However, lymph node metastases may be seen in 15% to 30% of patients with low-grade cancer. The standard treatment is hysterectomy [16-18]. Morcellation procedures are contraindicated if there is clinical suspicion of uterine sarcoma. It worsens the prognosis, given that preoperative diagnosis is difficult and unusual. The diagnosis is frequently made after histological study of the surgical specimen. In these cases, it is advisable to perform an imaging study to exclude metastatic disease, since more than 30% of these patients present metastatic disease in the liver, lungs, or upper abdomen [18,19]. The low incidence and the great histological, biological, and clinical heterogeneity of uterine sarcomas and of all primary mesenchymal uterine neoplasms, in general, have prevented randomized studies from being carried out. A minimum follow-up of 10 years is recommended for most sarcoma types since most sarcoma recurrences occur during the first three years after treatment and although their early detection is not clear to influence overall survival, the trend is to investigate early occurrence [20,21].

## Conclusions

It is a rare type of uterine tumor, with a great malignant potential and a poor prognosis, mainly due to the extent of the disease and its rapid progression. So, the survival rate depends on the stage of the disease at the time of diagnosis, which indicates the need to prevent advanced stages, especially in third world countries, and to continue research in search of a treatment that improves the prognosis of patients.
